# Engaging Female Community Health Volunteers (FCHVs) for cardiovascular diseases risk screening in Nepal

**DOI:** 10.1371/journal.pone.0261518

**Published:** 2022-01-06

**Authors:** Lal B. Rawal, Yuewen Sun, Padam K. Dahal, Sushil C. Baral, Sudeepa Khanal, Abriti Arjyal, Shraddha Manandhar, Abu S. Abdullah

**Affiliations:** 1 School of Health, Medical and Applied Sciences, College of Science and Sustainability, Central Queensland University, Rockhampton, Australia; 2 Physical Activity Research Group, Appleton Institute, Central Queensland University, Rockhampton, Australia; 3 Translational Health Research Institute, and School of Social Sciences, Western Sydney University, Penrith, Australia; 4 Global Health Institute, Duke Kunshan University, Jiangsu, China; 5 HERD International, Kathmandu, Nepal; 6 School of Public Health, Bielefeld University, Bielefeld, Germany; 7 Duke Global Health Institute, Duke University, Durban, NC, United States of America; 8 Boston University School of Medicine, Boston Medical Center, Boston, MA, United States of America; Bangalore Baptist Hospital, INDIA

## Abstract

**Introduction:**

Non-Communicable Diseases (NCDs) have become the leading public health problems worldwide and the cardiovascular diseases (CVDs) is one of the major NCDs. Female Community Health Volunteers (FCHVs) in Nepal are the key drivers to implementing frontline health services. We explored the potential for engaging FCHVs for CVD risk screening at the community level in Nepal.

**Methods:**

We used multiple approaches (quantitative and qualitative) for data collection. The trained FCHVs administered CVD risk screening questionnaire among 491 adults in rural and urban areas and calculated the CVD risk scores. To maintain consistency and quality, a registered medical doctor also, using the same risk scoring chart, independently calculated the CVD risk scores. Kappa statistics and concordance coefficient were used to compare these two sets of risk screening results. Sensitivity and specificity analyses were conducted. Two focus group discussions among the FCHVs were conducted to determine their experiences with CVD risk screening and willingness to engage with CVD prevention and control efforts.

**Results:**

The mean level of agreement between two sets of risk screening results was 94.5% (Kappa = 0.77, P<0.05). Sensitivity of FCHV screening was 90.3% (95% CI: 0.801–0.964); and the specificity was 97% (95% CI: 0.948, 0.984). FCHVs who participated in the FGDs expressed a strong enthusiasm and readiness to using the CVD risk screening tools. Despite their busy workload, all FCHVs showed high level of motivation and willingness in using CVD risk screening tools and contribute to the prevention and control efforts of NCDs. The FCHVs recommended needs for providing additional training and capacity building opportunities.

**Conclusion:**

We conclude that there is a potential for engaging FCHVs to use simple CVD risk screening tools at the community level. The findings are promising, however, further studies engaging larger number of FCHVs and larger population would warrant feasibility of such tools within the existing healthcare systems in Nepal.

## Introduction

Non-Communicable Diseases (NCDs) have become the leading public health problems worldwide [1–4]. In 2016, of the 57 million death occurred worldwide, 71% death were caused by NCDs [5]. This burden is alarming in low- and middle-income countries (LMICs), where over 85% of premature deaths and 78% of all NCD death occur [6]. The major NCDs responsible for these deaths are cardiovascular diseases (CVDs) (44% of all NCD deaths); cancers (22%); chronic obstructive respiratory diseases (COPD) (9%); and diabetes (4%) [5]. Evidence from the Global Burden of Diseases (GBD) study shows that CVDs caused more than 17.6 million deaths globally in 2016 [7] and over 18.6 million deaths in 2019 [1]. The prevalent cases of total CVD nearly doubled from 271 million in 1990 to 523 million in 2019 [1]. This burden is affecting not only the high-income countries (HICs), but also the LMICs, which are facing burden of both infectious diseases and NCDs [8].

Like other LMICs, Nepal in recent years is witnessing increasing burden of CVDs [9–12]. In 2016, CVDs accounted for 28.1% of total mortality, which was the number one leading cause of death in Nepal [7]. Compared to increased disease burden, CVDs receive disproportionately less attention and resource allocations within the Nepalese health system [13]. The awareness and treatment rate for hypertension and other chronic conditions in Nepal is alarmingly low [14]. For example, a study in Kathmandu reported that only 1/3 of people with hypertension were aware of their condition; among those who were aware, 23.5% received treatment [15]. Faced with the surging burden of CVDs and the limited numbers of health professionals in the country, more innovative approaches are needed to address the problem of CVDs. Evidence shows that the trained Community Health Workers (CHWs) could perform lifestyle interventions, improve medication adherence and manage various chronic conditions in community settings [16–19].

The increasing problem of NCDs mortality and morbidity in Nepal poses threat to not only worsening the health situation, but also affects overall socioeconomic development of the country, challenging Nepal’s ambition of achieving Universal Health Coverage and Sustainable Development Goals (SDGs) by 2030 [20]. The impact of NCDs on national economy, communities, family and the individual itself is unbearable [21, 22] and this depletes health resources in the country. Given the importance of prevention and control of NCDs, the Government of Nepal in recent years has taken policy and program initiatives including formulation of National Health Policy 2016/17; Multi-sectoral Action Plan for the Prevention and Control of NCDs (2014–2020); Control of alcohol and tobacco products; and scaling up Package for Essential Non-communicable diseases prevention and control (PEN) etc. [23]. In recent years, primary prevention of NCDs at population level has got prime attention, however several barriers exist including lack of trained human resources, unavailability of adequate health services at primary care level, lack of adequate supplies and logistics, and recording and reporting of health services etc. [19, 24, 25].

The Female Community Health Volunteers (FCHVs), introduced in 1988 by the Government of Nepal, are the frontline CHWs in Nepalese healthcare system and trusted by the community members [26]. The number of FCHVs in Nepal has reached over 51,000 as of end 2020. They are mainly engaged in delivering communicable diseases, reproductive health, maternal and child health, expanded program of immunization, family planning, and HIV prevention and control programs in Nepal [27]. FCHVs in Nepal have been playing important roles to implementing a range of health programs at the community level. They are also playing pivotal role in promoting and linking the community with the health care providers. FCHV-led programs have achieved many successes in areas of maternal and child health [28]. A few studies in the past decade have explored potential to engaging CHWs including FCHVs in NCDs prevention and control in Nepal [17, 19, 29], however, those studies did not explore whether FCHVs could be provided with the appropriate training to deliver NCDs prevention services (i.e. perform CVD risk screening). This study aimed to addressing the evidence gap in terms of engaging trained FCHVs to perform CVD risk screening at the community level in Nepal.

## Methods

This study used multiple approaches and was conducted in 3 phases during July and August 2017. The first phase involved selection and training of FCHVs to use CVD risk scoring chart as recommended by WHO [30]. The WHO recommended CVD risk screening chart is simple, easy to administer, widely used and recommended to use for LMICs [30]. The second phase was a quantitative assessment of FCHV’s capacity to conduct community-based cardiovascular risk screening and the third phase involved a qualitative study and included two focus group discussions (FGDs) among the FCHVs to investigate FCHVs’ training and fieldwork experiences during the study.

### Study site and FCHV (trainees) selection

This study was carried out in two purposively selected communities (one in urban and one in rural areas) of Lalitpur district, Nepal. Lalitpur is one of the best performing districts within the Ministry of Health and Population. FCHVs in this district have been playing significant roles in achieving better health outcomes compared to other districts. The study sites in this district were chosen, following the consultation meeting with Lalitpur District Public Health Office, and Ministry of Health and Population, Nepal. The selection of FCHVs was made through the discussion with Lalitpur District Public Health Office, Ministry of Health and Population Nepal. Four key criteria were used for recruiting FCHVs, including (i) working as a FCHV in the study sites; (ii) having completed at least 8^th^ grade of education or above; (iii) older than 18 years old; and (iv) voluntary willingness to participate in this study. Five FCHVs were purposively selected from each study site.

### Training and setting

The training to FCHVs was conducted by a Health Assistants (HA)/ or Community Medical Assistants (CMA) in Nepali language. The HA or CMA are community level health workers, who have certificate level training in general medicine, and they perform as an In-charge of community level health facility in Nepal [19]. There were two main focused areas in the training session including (i) a didactic training; (ii) an operational training. In the process of developing training manual and fieldwork handbook, a range of published literature were searched and reviewed. The PEN package for NCDs prevention and control, as recommended by WHO guided us as key reference to training contents [31]. All training materials were translated from English to Nepali, then back translated to English.

The didactic part of the training focused on improving knowledge on CVDs and health research ethics while involving human participants. The CVD related knowledge session covered the common NCD definitions, symptoms, and risk factors. FCHVs were also trained to provide evidence-based lifestyle change health education and recommendations to community members. The research ethics session was about the ethical principles and related issues while collecting data in communities.

The operational aspect of the training included how to measure blood pressure using the digital blood pressure measurement device, and CVD risk score calculation. After the training, a mock session was conducted to assess FCHVs’ ability of collecting reliable data using questionnaires. FCHVs who could not perform well during the mock session (2 FCHVs), additional training sessions were provided to help them qualify and ensure high level of confidence.

### Ethics approval

This study received ethics approval from Nepal Health Research Council, Kathmandu, (Ref#: 2444) and Institutional Review Committee of Duke Kunshan University, Suzhou, China (Ref#: FWA00021580). All participants including the community members and FCHVs provided written consent prior to participating in the study.

### CVD risk screening

Trained FCHVs visited target households using door-to-door visit to recruit eligible community participants in their own catchment area. The inclusion criteria were residing in the community; aged between 35–74 years old; no previous history of diagnosed CVD, including established coronary heart disease, cerebrovascular disease and peripheral vascular disease. Upon obtaining written informed consent, the FCHVs collected data from the eligible participants using the WHO recommended CVD risk scoring chart [30].

A CVD risk factor questionnaire collected data on age, gender, smoking habits, blood pressure and diabetes status. The participants self-reported their date of birth, diabetes status, and smoking behavior. Age was calculated from reported birth date. Data on diabetes status was collected using self-reported information. For the smoking behavior, all current smokers and those who quit smoking within past one year were considered as smokers. Blood pressure was measured using OMRON electronic blood pressure monitor (HEM-8712). Each CVD risk assessment lasted 30–45 minutes. During the fieldwork, the study coordinator maintained regular communication via phone calls with FCHVs and visited study sites and addressed the challenges faced by the FCHVs during the data collection process. Upon data collection, each community members were provided with a brief health education on importance of maintaining healthy lifestyles and a brochure containing information on CVDs risk and lifestyle interventions approaches to reduce potential CVD risk.

### CVD risk score calculation

The FCHVs and a health professional independently calculated cardiovascular risk score using the data collected by the FCHVs. The CVD risk factor questionnaires were de-identified by researchers before the health professional used them to calculate cardiovascular risk score. We estimated 10-year CVD risk using the 2019 WHO cardiovascular risk prediction charts [30, 32]. The prediction charts provide the 10-year risk of a fatal or non-fatal major cardiovascular event, such as myocardial infarction or stroke, based on age, sex, BP, BMI, smoking status, total blood cholesterol and the presence or absence of diabetes mellitus. In this study, we did not measure Cholesterol level and WHO CVD risk non-laboratory-based charts developed for south Asia region was used.

The prediction chart grades CVD risk using the following categories: age (1: 40–44 years; 2: 45–49 years; 3: 50–54 years; 4: 55–59 years; 5: 60–64 years; 6: 64–69 years; 7: 70–74 years), sex (male and female), smoking (smoker or non-smoker), systolic BP (SBP; <120 mmHg, 120–139, 140–159, 160 to <180 and ≥180) and BMI (<20, 20–24, 25–29, 30–35, and ≥35). The risk categories for 10-year combined acute myocardial infraction and stroke (fatal and non-fatal) include: <5%, 5% to <10%, 10% to <20%, 20%-<30% -15% and ≥30%.

### Qualitative assessment of FCHV’s experience using focus group discussion

After FCHVs completed their data collection and CVD risk scoring, two focus group discussion were conducted: one in urban site and another in rural site; each group consisted of 5 FCHVs. The purpose of this FGD was to gather FCHV’s opinion regarding the CVD risk scoring in their catchment areas, their experience of undertaking risk scoring including the difficulties they encountered, and recommendations for future use.

### Data analysis

Each FCHV conducted an average 50 CVD risk screening, with two FCHVs conducting 52 each. A total of 504 cases were screened for 10-year CVD risk assessment. Due to incomplete data (6 cases) and not meeting the inclusion criteria (7 cases), a total of 13 cases were excluded from the data analysis. Therefore, 491 screening cases were included for quantitative data analysis. Quantitative data analyses were performed using STATA 15. The significance level was set at 5% level. Kappa concordance statistics was used to assess the agreement between FCHV’s CVD risk screening results and doctor’s results. Further, concordance coefficient was used to analyze FCHVs’ performance as a group. The comparison was made between rural and urban FCHVs. Sensitivity and specificity test was also conducted to assess FCHV’s ability of screening CVD risk in their catchment. The distribution of risk factors of CVDs was reported for the population of this study. The comparison of risk factors distribution was made between rural and urban community. The qualitative data analyses involved iterative thematic analysis of transcripts. NVivo 11 software was used to facilitate the qualitative data analysis process.

## Results

The findings of the study are presented according to the study designs including quantitative and qualitative study designs.

### A. Findings from the quantitative approach

#### A1. FCHV characteristics

Ten FCHVs were purposively recruited in this study, 5 from the rural site and another 5 from the urban site. The overall age ranged between 38 and 49 (mean 43.4). The range of duration of serving as a FCHV was between one year and 24 years, with a median age 14 years of serving and over 70% FCHVs worked more than 10 years. Majority of them (80%) graduated grade 10 schooling, one graduated grade 8 and 1 graduated grade 5. The FCHVs from the urban site met all selection criteria. However, due to the limited number of eligible FCHVs in rural site, we lowered the educational requirements for one experienced FCHV who had a fifth grade education but was equally competent as other FCHVs.

#### A2. FCHV’s capacity for CVD risk screening (using Kappa Concordance test)

The results from Kappa Concordance test are shown in Table 1. The overall mean level of agreement between FCHVs’ and doctor’s risk screening score was 94.5% (Kappa = 0.77, P<0.05, 95% CI 0.705–0.835). Agreement level for FCHVs from the rural community was 93.5% (Kappa = 0.7759, P<0.05), whereas urban community 95.5% (Kappa = 0.7568, P<0.05, 95% CI 0.685–0.867). The level of agreement ranged between 82.6% and 100%. Overall, the FCHVs from the urban community performed better than the FCHVs from the rural community (agreement level 95.51% versus 93.50%, respectively).

**Table 1:** Capacity assessment (Direct degree of agreement and kappa statistic) by comparing FCHVs’ and doctor’s screening results.

FCHV	Agreement	Kappa (95% CI)	p-value
1	92.0%	0.781 (0.576, 0.987)	<0.001
2	98.0%	0.931 (0.779, 1.083)	<0.001
3	96.0%	0.859 (0.677, 1.042)	<0.001
4	82.6%	0.427 (0.146, 0.707)	<0.001
5	98.0%	0.901 (0.569, 1.233)	<0.001
6	95.8%	0.584 (0.105, 1.064)	<0.001
7	89.8%	0.543 (0.183, 0.903)	<0.001
8	95.9%	0.841 (0.669, 1.013)	<0.001
9	96.0%	0.778 (0.394, 1.162)	<0.001
10	100.0%	1.000 (-)	<0.001
Rural FCHVs	93.5%	0.776 (0.685, 0.867)	<0.001
Urban FCHVs	95.5%	0.757 (0.603, 0.910)	<0.001
**Overall**	**94.5%**	**0.770 (0.705, 0.835)**	**<0.001**

#### A3. Concordance correlation coefficient

The concordance correlation coefficient for FCHV’s CVD risk screening test was 0.897 (95% CI: 0.880, 0.914), which indicates good level of precision and accuracy of FCHV’s CVD risk screening test. The concordance coefficient in rural site was 0.877 (95%CI: 0.849, 0.905); whereas in urban site was 0.919 (95% CI: 0.900, 0.939).

**Fig 1:**
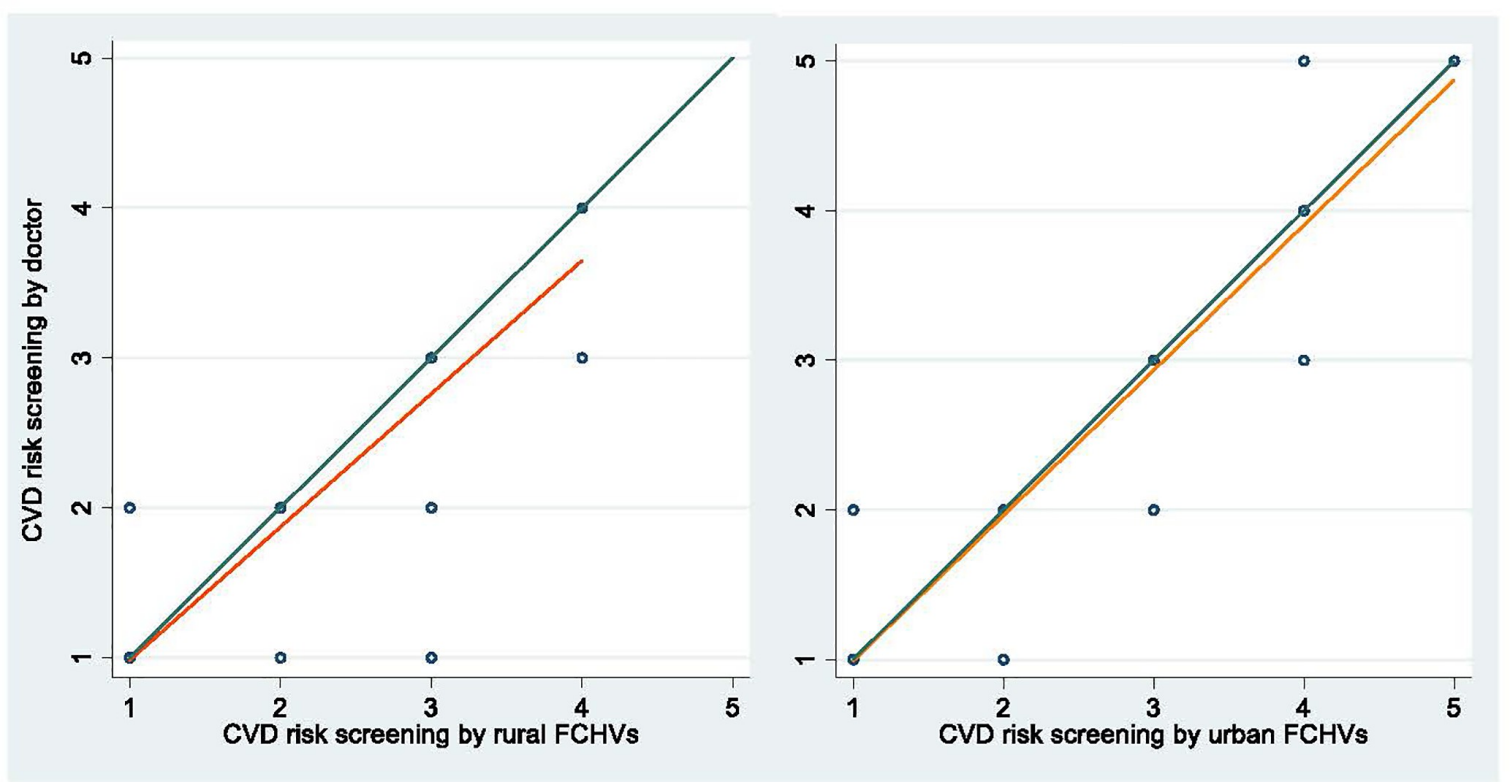
Concordance correlation coefficient for rural and urban sites.

Fig 1 provides concordance plot for rural site and urban site. The green lines in both graphs indicate perfect concordance; the orange lines are the actual plot for FCHVs’ performance at rural and urban sites. The closer to the green line shows a better concordance.

**Fig 2:**
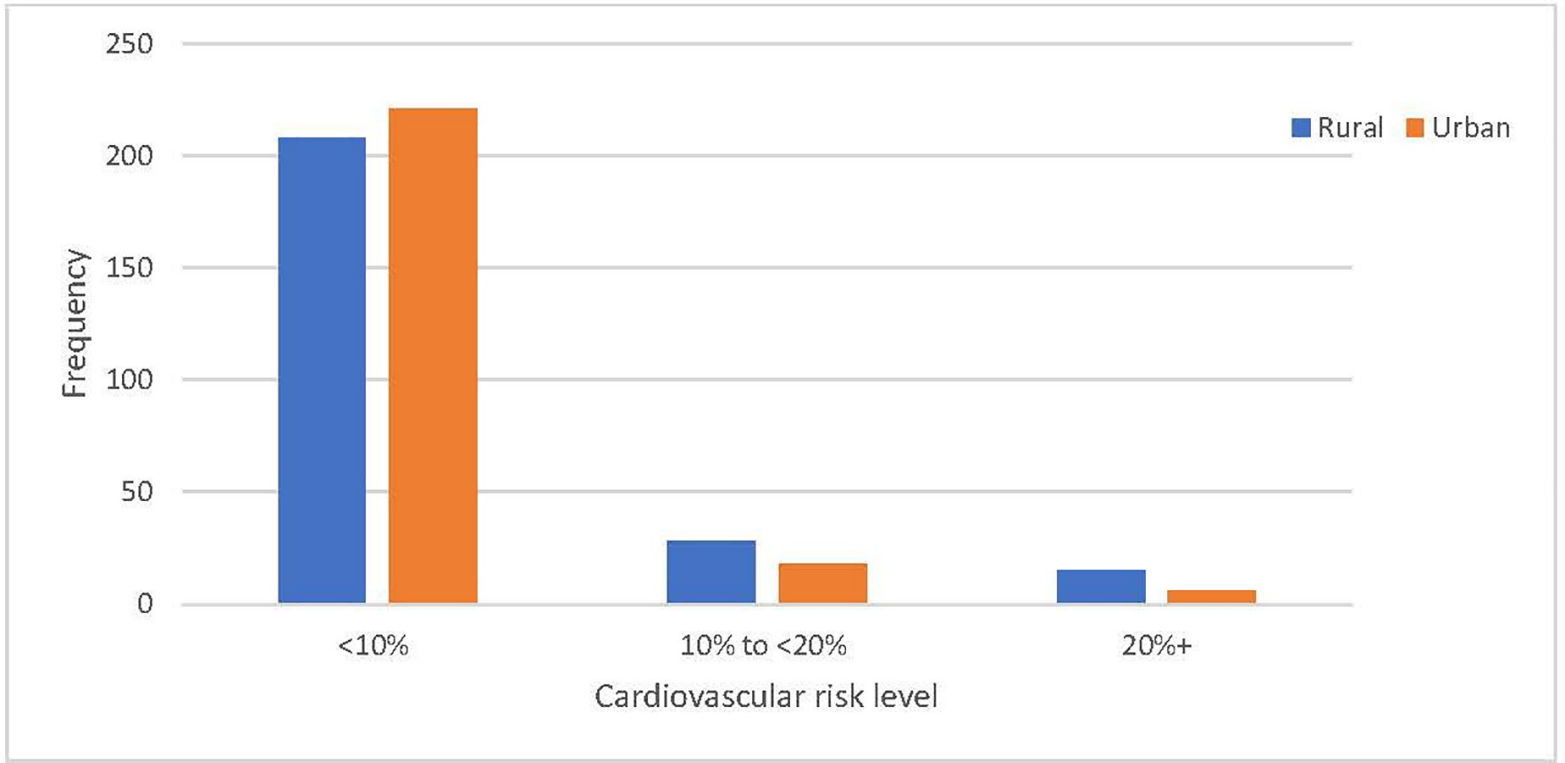
Comparison of distribution of cardiovascular risk score by region.

As shown in Fig 2, the urban FCHVs’ screening tests are slightly better in terms of concordance with the gold standard compared to rural FCHVs’ screening test scores.

#### A4. Sensitivity and specificity of FCHV’s CVD risk scoring

The sensitivity and specificity analyses of FCHV screening test scores were calculated to explain the reliability compared with doctor’s CVD risk screening scores, which was treated as gold standard in this study. The sensitivity test scores of FCHV screening test was 90.3% (95% CI: 80.1%, 96.4%); and the specificity was 97% (95% CI: 94.8%, 98.4%). The detailed data used for calculating sensitivity and specificity analyses is provided in Table 2.

**Table 2:** Comparison of CVD screening test between FCHVs and medical doctor.

FCHV’s CVD risk screening	Doctor’s CVD risk screening		Total
	Elevated risk (≥10%)	No elevated risk (<10%)	
Elevated risk (≥10%)	56	13	69
No elevated risk (<10%)	6	416	422
Total	62	429	491

Note: The cardiovascular risk level is treated as a dichotomous variable here for calculating specificity and sensitivity of FCHV screening test. Cardiovascular risk higher than 10% is regarded as elevated cardiovascular risk in following 10 years; cardiovascular risk lower than 10% is regarded as no elevated cardiovascular risk. Doctor’s screening result is treated as gold standard.

#### A5. CVD risk score distribution among study population

A total of 491 participants were screened (246 from the rural community and 245 from the urban community). The mean age of participants was 50 years old; 71.5% were female; 10.2% reported a history of diagnosed diabetes; 14.7% had elevated blood pressure and 18.5% were current smokers. According to the doctor’s screening test results, 87.4% of the participants had a risk less than 10% and 12.6% cases had a risk greater than 10% (i.e. a relatively high risk of developing cardiovascular event(s) in the following 10 years). The cardiovascular risk was slightly higher in the rural participants compared to urban cohort, and this difference was not statistically significant (P = 0.105) (Table 3).

**Table 3:** Distribution of cardiovascular risk level in the rural and urban community.

CVD risk level	Rural	Urban	Total	P-value
<10%	208	221	429	0.105 (fisher exact)
10% to <20%	28	18	46	
20% to <30%	8	3	11	
30% to <40%	2	1	3	
>40%	0	2	2	
Total	246	245	491	

#### A6. Prevalence of cardiovascular disease risk factors

The overall prevalence of self-reported diabetes and elevated blood pressure was 9.0% and 14.7%, respectively. As shown in Table 4, there was a significant difference of self-reported diabetes prevalence between rural (7.3%) and urban (13.1%) participants (P = 0.0035). However, the prevalence of hypertension was higher among the rural participants (18.3%), compared to urban cohorts (11.0%) (P = 0.023). The overall smoking prevalence was 18.5% with no significant difference between rural and urban participants.

**Table 4:** Distribution of three major CVD risk factors.

Cardiovascular risk factors	Rural	Urban	Chi^2^	P-value
Self-reported diabetes				
Yes	18	32	4.4282	0.0035
No	228	213		
High Blood pressure				
Yes	45	27	5.1877	0.023
No	201	218		
Current smoker				
Yes	46	45	0.0090	0.925
No	200	200		
Total	246	245		

### B. Qualitative findings obtained from the FCHV’s involved for field data collection

The qualitative findings obtained from FCHVs who participated in the training and were involved in field data collection, is structured in following four themes:

#### Theme 1: Perceived workload

FCHVs did not feel burdensome to the works they were conducting. The FCHVs in Nepal are involved in providing education and support to promote safe motherhood, child health, family planning, expanded program of immunization, and health education for improving healthy behavior. In addition, community members receive advice for basic health services such as oral rehydration solution, family planning devices when needed. FCHVs would also walk in the community from time to time and respond to any health services related queries from community members. All FCHVs thought that these services were manageable, however, the situation may become difficult when FCHVs need to deal more with their own household works. One FCHV said:

“*We do not feel much work burden*. *It has been 17 years that we are providing services*. *It was a bit difficult before when we were daughters-in-law*. *We had to give our time in our home too*. *Now we have got experience and I am so happy that we can provide the service from our own house*.*”–FCHV*, *rural community*

#### Theme 2: Motivation for FCHVs’ works

The FCHVs in Nepal are voluntary community health workers working within the Ministry of Health and Population, Government of Nepal. Comparing to full-time CHWs in other countries, internal motivation plays an important role for FCHVs in Nepal. Most FCHVs thought that the respect and recognition they receive from the community members were the main drivers to keep them motivating to serve their communities. One FCHV said:

“*When we visit the community wearing the uniform of Nepal Government*, *as soon as they see us they [community people] say what is today’s program*? *Is there Vitamin A [distribution]*, *are you vaccinating children*?*”- FCHV urban community*.

Another FCHV said:

“*And even when we don’t wear dress [FCHV dress] and walk*, *the ones who know us ask “Oh FCHV didi [sister]*, *where are you going now” and it makes us happy*.*”–FCHV urban community*.

Regarding the financial motivation, almost all FCHVs said that financial compensation has not been a priority. However, they opined that having financial support provided would certainly further motivate them and ensure long-term engagement. Some FCHVs mentioned that non-financial support such as uniform, lunch and snack, provision of health care etc. would also incentivize them for voluntarily supporting in their community. One FCHV said:

“*It would be good if the Government provides us some materials to provide the services to community people and also to organize meetings would be enough to motivate them for the works they have been doing”—FCHV rural community*.

#### Theme 3: Attitudes towards community-based cardiovascular risk screening

FCHVs were enthusiastic when asked about their attitudes towards community-based cardiovascular risk screening—main component of the study. Nearly all FCHVs noticed the growing number of patients with chronic conditions in their community and expressed their desire to help community members. One FCHV said,

“*Cardiovascular diseases can be reduced*. *Those who are already suffering cannot be cured*. *But we can prevent those who have not suffered yet*.*”*

Some FCHVs mentioned that they had been wanting such a training (the training they received for this study) since a long time. One FCHV said:

“*We have been asking for a long time with the health post*, *DPHO (District Public Health Office) to give us a BP set [BP monitoring set] and training to how to measure BP*. *However this has not yet happened*. *In this particular project*, *it feels good that we have received training*, *BP set and training for measuring BP*. *This feels really good and happy*.*”- FCHV rural community*.

#### Theme 4: Difficulty encountered in performing CVD risk screening in their community

Most FCHVs reported no major difficulty during their fieldwork. However, there were few minor issues they faced, such as question on reliability of the digital BP devices, types and dose of hypertension and diabetes medications that were asked by the community members. One FCHV reported that it was hard to explain the meaning of 10-year cardiovascular risk to the community participants, indicating more training was needed in this particular aspect to enhance understanding of CVD risk and risk score assessment.

## Discussion

To the best of our knowledge, this is the first study ever been conducted to potentially engage FCHVs to perform CVD risk screening in Nepal. The findings proved that FCHVs with secondary school level education could effectively perform community-based CVD risk screening upon receiving training. After the three-day training, FCHVs could accurately identify adults with high risk of developing CVDs in the following 10 years using the non-invasive CVD risk scoring chart. The results of the CVD risk screening by FCHV showed high level of sensitivity and specificity while comparing with the ones performed by the registered medical doctors. The kappa concordance test results showed overall high level of sensitivity and specificity. No significant difference between the CVD risk scores of rural and urban FCHVs was observed. Evidence suggest that determining the people at risk of developing CVD can help developing cost-effective intervention approaches to prevent development of CVDs [33, 34] and minimize disability and mortality due to CVD [35, 36].

We found that 13% of participants had over 10% risk scores of possibly developing CVDs in the following 10 years. This level of CVD risk is slightly lower than the findings in other past studies [37, 38]. A CHW-led CVD risk screening study by Gaziano et. al., reported that 22.4% participants had a 5-year cardiovascular risk greater than 10% [37]. This difference could be explained by the different risk scoring tools used in the Gaziano study. In this study, we used the 10-year CVD risk scoring chart as recommended by WHO [30], while the Gaziano et al., used a self-developed 5-year CVD risk scoring chart [37]. The CVD risk scores as identified in this study echoes the increasing trends of NCD risk i.e. high prevalence of smoking, diabetes and hypertension in Nepal. In our study, the prevalence of self-reported diabetes (13.1% in the urban site and 7.3% in the rural site) was higher than the nation-wide estimated prevalence [9] and the one estimated by International Diabetes Federation [39]. A recently conducted nationwide NCD study in Nepal showed overall prevalence of diabetes at 8.0% [9, 19]. In our study, the prevalence of elevated blood pressure was 45% in rural and 27% in urban sites. These findings are also slightly higher than the national average of 24.5% [9] and 18% [40] in rural and urban areas, respectively.

The FCHVs in Nepal are the key drivers to implementing frontline health services, in particular the programs related to the prevention and control of communicable diseases, immunization services, reproductive health, maternal and child health, HIV/AIDS prevention and care, and nutrition promotion [27]. Given the increasing burden of NCDs including CVDs in the country, and the fact that the FCHVs are willing to contribute to the NCDs related programs as shown in this study, there is a potential for engaging FCHVs for NCDs prevention and control efforts, particularly for CVDs prevention and control. Further, given the increasing use and effectiveness of technologies, such as smart phone, tablets, and other mobile phone devices, there is a need and possibilities for using such technologies by FCHVs for CVD risk screening, real time reporting, providing health education and counselling, referral and follow up etc. in the case of Nepal.

To ensure quality services and consistent services across FCHVs in the country, they would require additional trainings focused on NCDs prevention and control in general and specifically focused on CVD risk screening at the community level. Given the literacy and education level of FCHVs across Nepal is not uniform, the training programs could be customized to fit their capacity and context. Community-based health literacy program will be more significant in prevention and management of chronic diseases [41]. Further, the health literacy initiative at the community level is essential to improving knowledge and understanding of symptoms of CVD and helping people to seek health services in a timely manner [42, 43]. Involvement of FCHVs in such programs would help minimizing the CVD risk at the community level and reduce NCDs burden in the long run. Consistent with the findings in other studies [29, 44], the current study supports the engagement of FCHVs in the NCDs prevention and control, however, further research is needed which would involve larger sample size with broader participation of FCHVs across Nepal.

Further, re-engineering the health workforce could be implemented along with re-structuring of the health systems including training of FCHVs in new skill sets and providing them with NCDs specific training focused on CVDs screening at community level, and providing health education and promotion to risk population [44]. Further, there is a need for developing a functional model to deliver NCD programs through re-vitalized primary healthcare approach with the involvement of FCHVs and other CHWs, who could essentially collaborate and co-ordinate the healthcare delivery. Adding to this, while the government of Nepal is grappling with the financial challenges for implementing NCD programs in the country, adopting a community-based approach (working along with different stakeholders) could be another avenue to plan and implement NCDs, more specifically CVDs related programs effectively.

### Strengths and limitations

This is the first study focused on training FCHVs in Nepal for CVD risk screening. The exploration and findings of this study provided valuable insights for further investigations of involving FCHVs in the prevention and control efforts of CVDs and other NCDs. However, this study has some limitations. First, the FCHVs who were involved in this study had relatively higher level of education (80% attained grade 10) compared to the national average (21%) [45] which may limit the generalizability of the study findings across the country. Second, we collected self-reported diabetes status data. Thich may over or underestimate the findings and CVD risk scoring. The WHO PEN recommends testing of urine sugar to replace the plasma glucose test for assessing CVD risk in very low resource settings [32]. Further, in the qualitative study, only 10 FCHVs who were involved in the CVD risk assessment participated in the two FGDs. The findings obtained from these FGDs may not be generalizable to the larger context of FCHVs in the country.

## Conclusions

The findings suggests that FCHVs could be trained to involve at the community level to use simple CVD risk screening tools. Further training and capacity building of FCHVs is essential, especially on NCDs prevention and control, CVD risk screening at community level and health promotion to prevent CVDs. Findings from this study are promising, however, further studies with larger sample size across the country is recommended.

## Supporting information

S1 Dataset (XLSX)
